# The relationship between urinary albumin to creatinine ratio and all-cause mortality in the elderly population in the Chinese community: a 10-year follow-up study

**DOI:** 10.1186/s12882-021-02644-z

**Published:** 2022-01-05

**Authors:** Anhang Zhang, Man Li, Jiaojiao Qiu, Jin Sun, Yongkang Su, Shuang Cai, Qiligeer Bao, Bokai Cheng, Shouyuan Ma, Yan Zhang, Shuxia Wang, Ping Zhu

**Affiliations:** 1 Medical School of Chinese PLA, Beijing, 100853 China; 2 Department of Geriatrics, The Second Medical Center & National Clinical Research Center for Geriatric Diseases, Chinese PLA General Hospital, 28 Fuxing Road, Beijing, 100853 China; 3 Department of Geriatric Cardiology, the second Medical Center, Chinese PLA General Hospital, Beijing, 100853 China; 4 Department of Outpatient, the first Medical Center, Chinese PLA General Hospital, Beijing, 100853 China

**Keywords:** Urinary albumin to creatinine ratio (UACR), All-cause mortality, Elderly, Albuminuria

## Abstract

**Background:**

In patients with diabetes and hypertension, proteinuria is independently associated with all-cause death. However, in the general population, urinary albumin to creatinine ratio (UACR) is less used to predict all-cause mortality. When the urinary albumin to creatinine ratio is within the normal range (UACR< 30 mg/g), the clinical relevance of an increased urinary albumin excretion rate is still debated. We studied the relationship between UACR and all-cause mortality in community populations, and compared UACR groups within the normal range.

**Methods:**

The participants were the inhabitants from the Wanshoulu community in Beijing, China. The average age is 71.48 years, and the proportion of women is 60.1%. A total of 2148 people completed random urine samples to determine the urinary albumin to creatinine ratio (UACR). The subjects were divided into three groups according to UACR: Group 1 (UACR< 10 mg/g), Group 2 (10 mg/g < UACR< 30 mg/g), Group 3 (UACR> 30 mg/g). We used Kaplan-Meier survival analysis and Cox regression model to verify the relationship between UACR and all-cause mortality.

**Results:**

At an average follow-up of 9.87 years (718,407.3 years), the total mortality rate were 183.4/1000. In the Cox proportional hazards model, after adjusting for possible confounders, those with normal high-value UACR (group 2) showed a higher all-cause mortality than those with normal low-value UACR (group 1) [hazard ratio (HR) 1.289, 95% confidence interval (CI) 1.002 ~ 1.659 for all-cause mortality]. Those with proteinuria (group 3) showed a higher all-cause mortality than those with normal low-value UACR (group 1) [hazard ratio (HR) 1.394, 95% confidence interval (CI) 1.020 ~ 1.905 for all-cause mortality].

**Conclusion:**

Urinary albumin to creatinine ratio is an important risk factor for all-cause death in community population. Even if it is within the normal range (UACR< 30 mg/g), it occurs in people with high normal value (10 mg/g < UACR< 30 mg/g), the risk of all-cause death will also increase.

## Background

With the acceleration of aging, when the age structure of the population becomes less young, tools for predicting the risk of all-cause mortality in the middle-aged and elderly have become more and more important in research, policy, and clinical practice [1–4]. Chronic kidney disease (CKD), defined as the presence of proteinuria and/or decreased renal function, is an /important risk factor for end-stage renal disease (ESKD), cardiovascular events and all-cause death [5]. In the general population, the association between proteinuria and all-cause death has received widespread attention. Albuminuria is highly prevalent among older adults, especially those with diabetes and hypertension [6–9]. It is associated with several chronic diseases [10]. However, its overall impact on the health of the elderly, as well as on all-cause mortality in the community, has not been studied in detail [11, 12]. The urinary albumin to creatinine ratio is a new and reliable method for monitoring the excretion of urine protein. The measurement of the urinary albumin to creatinine ratio can reliably reflect the amount of urine protein in 24 h. It is fast, simple and accurate. Urinary albumin to creatinine ratio has become a clinically qualitative and quantitative diagnostic index for proteinuria that can replace the traditional 24-h urine protein quantification [13, 14]. Studies have shown that high levels of urinary albumin excretion are associated with an increase in cardiovascular (CV) mortality, especially in patients with diabetes, and hypertension, as well as those with a history of cardiovascular disease, but there are few studies on the prediction of all-cause mortality in community populations [15–19]. In addition, in healthy people (UACR less than 30 mg/g), the urinary albumin to creatinine ratio as a risk indicator is controversial in predicting all-cause death in healthy people [20]. Their previous relationship and degree of association have not yet been specifically studied [2, 17, 21]. The few published studies on the urinary albumin to creatinine ratio and all-cause mortality are aimed at smaller populations, relying on retrospectively collected data, or the population is a special population with underlying diseases such as diabetes and hypertension [6–9, 22–24]. Therefore, in this study, we selected a relatively large cohort from the community population, followed up for 10 years, and studied the relationship between urinary albumin to creatinine ratio and all-cause mortality from multiple levels.

## Methods

### Subjects

**Fig. 1:**
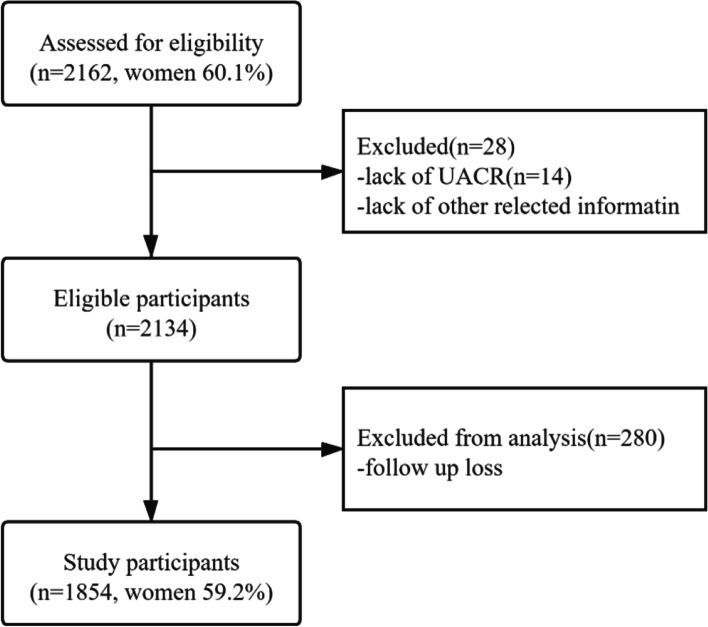
Flow chart for the selection of study participants

Sampling and research methods were reported elsewhere [25, 26]. The previous study used a two-stage stratified clustering sampling method was used to conduct this population-based cross-sectional survey in participants living in Wanshoulu community, a representative urban residential area of Beijing. This study is based on a large-scale cohort study conducted by previous community populations. The average age is 71.48, the standard deviation is 6.77, and the proportion of women is 60.1%. Recruitment of the Wanshoulu community started in 2010, and a follow-up survey was conducted on the participants. Figure 1 shows the recruitment process of the study population.

### Data collection

Men and women filled out detailed baseline health and lifestyle questionnaires, and 2162 people participated in a health check conducted by trained nurses using standard procedures. At the time of consultation, a random urine sample was randomly selected to determine the urinary albumin to creatinine ratio (UACR). Information about drinking and smoking status, diabetes, hypertension, coronary heart disease history, NIHSS stroke scale, MMSE simple intelligence scale, ADL daily activity scale and other information were obtained from baseline health data and lifestyle questionnaires. The body mass index (BMI) is calculated by dividing the weight (Kg) by the square of the height (m), measuring the height with a stand-alone distance meter, and calculating the weight with an electronic scale. After the participant sat and rested for 5 min, the blood pressure was measured using a sphygmomanometer. The systolic and diastolic blood pressure were measured twice, and the average of the two was used for analysis. Hypertension is defined as high blood pressure or systolic blood pressure > 140 mmHg or diastolic blood pressure > 90 mmHg as diagnosed by a doctor. Routine blood tests for fasting blood glucose, 2 h postprandial blood glucose, glycosylated hemoglobin, total serum cholesterol, high-density lipoprotein cholesterol, low-density lipoprotein cholesterol and triglycerides were measured on an automatic biochemical analyzer. All on-site urine samples were taken to determine the concentration of urinary albumin (mg/l) and the concentration of urinary creatinine (g/l), and calculate the UACR (mg/g). All biochemical analyses were performed in the Department of Biochemistry of the Chinese People’s Liberation Army General Hospital. Among the subjects, 1854 agreed to participate in the telephone follow-up or signed a written informed consent for community on-site follow-up, which constituted our study population. Regarding age and gender, there is no difference between responders and non-responders. This study was ethically approved by the Ethics Committee of the Chinese People’s Liberation Army General Hospital.

### Study design

Our observations add to more and more evidence, and they challenge the idea that UACR< 30 mg/g means albumin excretion is “normal” [27]. Our research is more focused on “normal” urine albumin excretion, so we divide the urine protein-creatinine ratio into three groups, divide the range of proteinuria with a limit of 30 mg/g, and then divide the “normal” urinary albumin to creatinine ratio by 10 mg/g is divided into two groups. Analyze and compare the relationship between urinary albumin to creatinine ratio and all-cause death in the three groups [23, 28].

### Endpoints

Endpoint death cases were defined as indicator events that occurred between baseline and follow-up until December 31, 2020. During the follow-up period, the main concern was all-cause death. In these analyses, all-cause death is defined as death from any cause, and the cause of death comes from the results of telephone calls or on-site follow-ups provided by family members. For some members of the group whose telephone numbers were lost to follow-up or whose home address was changed, our staff sought help from Wanshoulu community staff and police officers from the population management archives office of Wanshoulu police station.

### Statistical analysis of data

All continuous data are expressed as mean ± standard deviation, while categorical data are expressed as absolute numbers and percentages (%). The Kolmogorov-Smirnoff test is used to check whether the data has a normal distribution. One-factor analysis of variance (ANOVA) test is used to analyze the baseline characteristics of the study populations. Post hoc multiple comparisons, the LSD test is used to assume that the variances are equal, and the Tamhane’s T2 test is used to assume that the variances are unequal. The chi-square test was used to compare the all-cause mortality of different groups after 10 years. The Kaplan-Meier method and log-rank test were used to compare cumulative probabilities. In the analysis of the Cox proportional hazards model, hazard ratio (HR) and 95% confidence interval (CI) were calculated after adjusting for age, gender, height, weight, BMI, waist circumference, systolic blood pressure, diastolic blood pressure, triglycerides, blood creatinine, blood uric acid, history of coronary heart disease, history of diabetes, history of drinking. A multivariate logistic regression model was established to show the relationship between UACR and all-cause mortality. In the cox regression analysis, the Omnibus test is used to test the model coefficients, and the results are analyzed by one-minus survival analysis function, hazard function, and LML function. In all hypothesis tests, the risk of the first type of error is set a priori as *P* < 0.05. All statistical tests are two-sided, and the significance level is α = 0.05. Use SPSS software (version 26.0) for statistical analysis.

## Results

### Baseline characteristics

Among the 1854 study populations, there were 229 people with a UACR greater than 30 mg/g, and the prevalence of proteinuria was 12.3%; 1149 people with a UACR less than 10 mg/g, accounting for 62.0% of the total; 476 people with a UACR of 10 mg /g to 30 mg/g, accounting for 25.7% of the total number of people. Women accounted for 59.2% of the population. One-factor analysis of variance (ANOVA) test is used to analyze the baseline characteristics of the study populations. Post hoc multiple comparisons, the LSD test is used to assume that the variances are equal, and the Tamhane’s T2 test is used to assume that the variances are unequal. Table 1 shows the baseline characteristics of the study population by study group.

**Table 1:** Baseline characteristics of the study population (all men and women), by categories of UACR

Parameter	Urinary albumin to creatinine ratio 0-10 mg/g (*N* = 1149)	Urinary albumin to creatinine ratio 10-30 mg/g (*N* = 476)	Urinary albumin to creatinine ratio > 30 mg/g (*N* = 229)	P
**Age (years)**	70.18 ± 6.87	72.26 ± 7.10	73.00 ± 6.74	< 0.001
**Sex (females %)**	55.0	66.6	65.1	< 0.001
**BMI (kg/m**^**2**^**)**	24.78 ± 3.26	25.16 ± 3.59	25.50 ± 3.83	0.001
**Waist (cm)**	87.69 ± 9.43	88.14 ± 9.04	89.69 ± 10.02	0.007
**Hip (cm)**	97.90 ± 8.34	98.61 ± 7.77	98.21 ± 8.20	0.077
**Systolic pressure (mmHg)**	135.62 ± 17.87	141.08 ± 20.65	145.97 ± 20.26	< 0.001
**Diastolic pressure (mmHg)**	76.46 ± 8.92	77.97 ± 10.62	78.78 ± 11.34	< 0.001
**FBG (fasting blood glucose) (mmol/L)**	5.89 ± 1.30	6.05 ± 1.56	6.66 ± 2.26	< 0.001
**2-h post-meal blood glucose (mmol/L)**	8.03 ± 3.19	8.23 ± 3.15	8.63 ± 4.13	0.031
**Glycosylated hemoglobin (%)**	5.96 ± 1.09	6.14 ± 1.23	6.54 ± 1.60	< 0.001
**Total cholesterol (mmol/L)**	5.22 ± 0.98	5.28 ± 1.03	5.29 ± 1.10	0.219
**High-density lipoprotein (mmol/L)**	1.42 ± 0.38	1.43 ± 0.37	1.39 ± 0.46	0.427
**Low-density lipoprotein (mmol/L)**	3.23 ± 0.83	3.23 ± 0.89	3.22 ± 0.93	0.896
**Triglyceride (mmol/L)**	1.61 ± 0.90	1.68 ± 0.77	1.86 ± 1.12	< 0.001
**Serum creatinine (umol/L)**	73.69 ± 16.24	71.29 ± 18.25	84.54 ± 43.46	< 0.001
**Blood uric acid (umol/L)**	309.32 ± 85.21	301.28 ± 93.74	326.25 ± 92.90	0.006
**NIHSS**	0.09 ± 0.40	0.16 ± 0.63	0.24 ± 0.94	0.008
**MMSE**	27.40 ± 2.84	26.29 ± 3.80	26.18 ± 4.22	< 0.001
**ADL**	99.24 ± 5.75	98.93 ± 5.39	98.55 ± 6.15	0.348
**Smoking (%)**	30.6	29.6	30.1	0.925
**Drinking (%)**	28.0	19.5	22.7	0.001
**CHD (%)**	20.9	23.5	35.8	< 0.001

*NIHSS* National Institutes of Health Stroke Scale, *MMSE* Minimum Mental State Examination, *ADL* Activity of Daily Living, *CHD* Coronary Heart Disease

### All-cause mortality

During the average follow-up period of 9.87 years (18,407.3 person-years of follow-up), 1854 subjects were followed up with 340 deaths (185 men and 155 women). The total population mortality, the rough all-cause mortality rates for males and females were 183.4/1000, 99.8/1000 and 83.6/1000, respectively. The mortality rate of men is slightly higher than that of women, which is consistent with the results of other studies in the region. Table 2 shows the comparison of all-cause mortality among the three groups according to the urinary albumin-creatinine ratio through the chi-square test.

**Table 2:** Chi-square test of all-cause mortality in different UACR groups

Survive crosstabulation					
			Survive		Total
			0	1	
Group	1	Count	981	168	1149
		% within group	85.4%	14.6%	100.0%
	2	Count	372	104	476
		% within group	78.2%	21.8%	100.0%
	3	Count	161	68	229
		% within group	70.3%	29.7%	100.0%
Total		Count	1514	340	1854
		% within group	81.7%	18.3%	100.0%

Group 1 (UACR< 10 mg/g), Group 2 (10 mg/g < UACR< 30 mg/g), Group 3 (UACR> 30 mg/g)

Survive 1 means All-cause deaths, Survive 0 means Number of people surviving

**Fig. 2:**
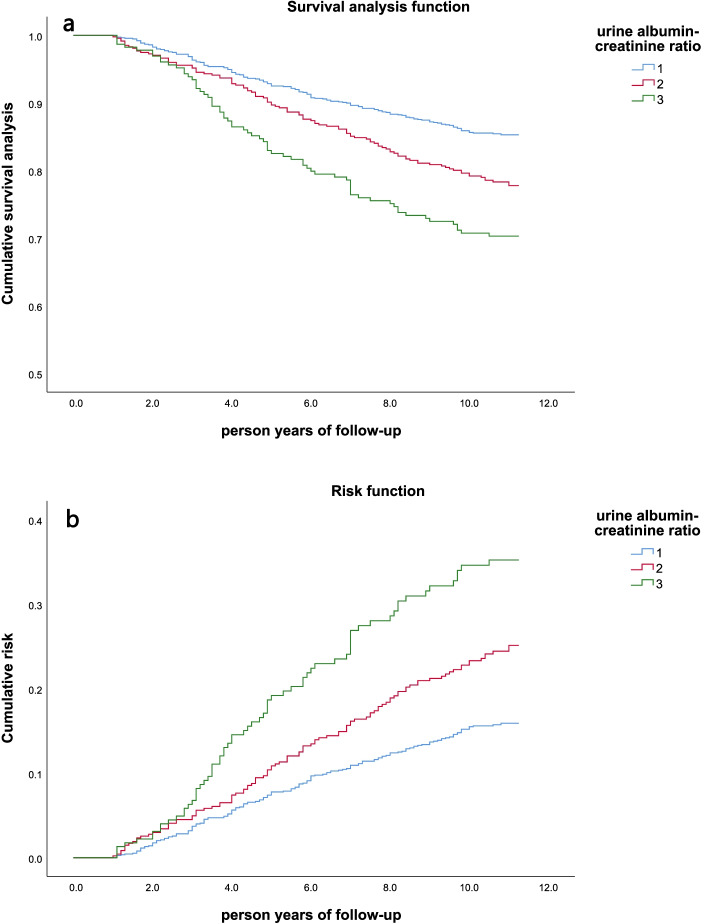
Kaplan-Meier survival curves of different UACR groups in the study population (*n* = 1854) classified by the ratio of urine albumin to creatinine. **a** Survival analysis function; **b** Risk function. Group 1 (UACR< 10 mg/g), Group 2 (10 mg/g < UACR< 30 mg/g), Group 3 (UACR> 30 mg/g)

As shown in Table 2, the mortality rate of people with proteinuria (Group 3) was 29.7%, which was higher than that of people without proteinuria. Among the two groups with normal urine protein, the mortality rate of the group with the higher UACR (Group 2) was 21.8%, which was higher than that of the group with the lowest UACR (Group 1). The higher the urinary albumin to creatinine ratio, the higher the mortality rate of the study population. Kaplan–Meier method and log-rank test were used to compare the cumulative probability. Figure 2 shows the Kaplan-Meier survival curves for different urine albumin-creatinine ratios. There is a significant dose-response relationship between UACR and all-cause mortality. The survival rate of the study population showed a significant downward trend with the increase of UACR (*p* < 0.001, tested by Log-Rank). It can also be seen from the Risk function graph that the greater the UACR, the greater the cumulative survival risk of the group.

### Mortality risk

In order to study the effect of urine albumin-creatinine ratio on all-cause mortality, we performed Cox proportional hazards model fitting on the data. In addition to UACR, the following variables that can be used to explain all-cause deaths and differences in the group in the baseline analysis are included in the regression analysis: age, gender, height, weight, BMI, waist circumference, systolic blood pressure, diastolic blood pressure, triglycerides, blood creatinine, blood uric acid, history of coronary heart disease, history of diabetes, history of drinking. After adjusting for the above variables that may affect survival, compared with Group 1 (UACR< 10 mg/g), Group 2 (10 mg/g < UACR< 30 mg/g) and Group 3 (UACR> 30 mg/g) have significant all-cause mortality increase. The Cox proportional hazard model was used to analyze multiple variables, which allows us to compare the effects of different variables on deaths from all causes. The results are summarized by the hazard ratio, by plotting the adjusted risk ratio as a function of UACR, and by plotting the causal specific cumulative correlation function of specific covariate values.

**Fig. 3:**
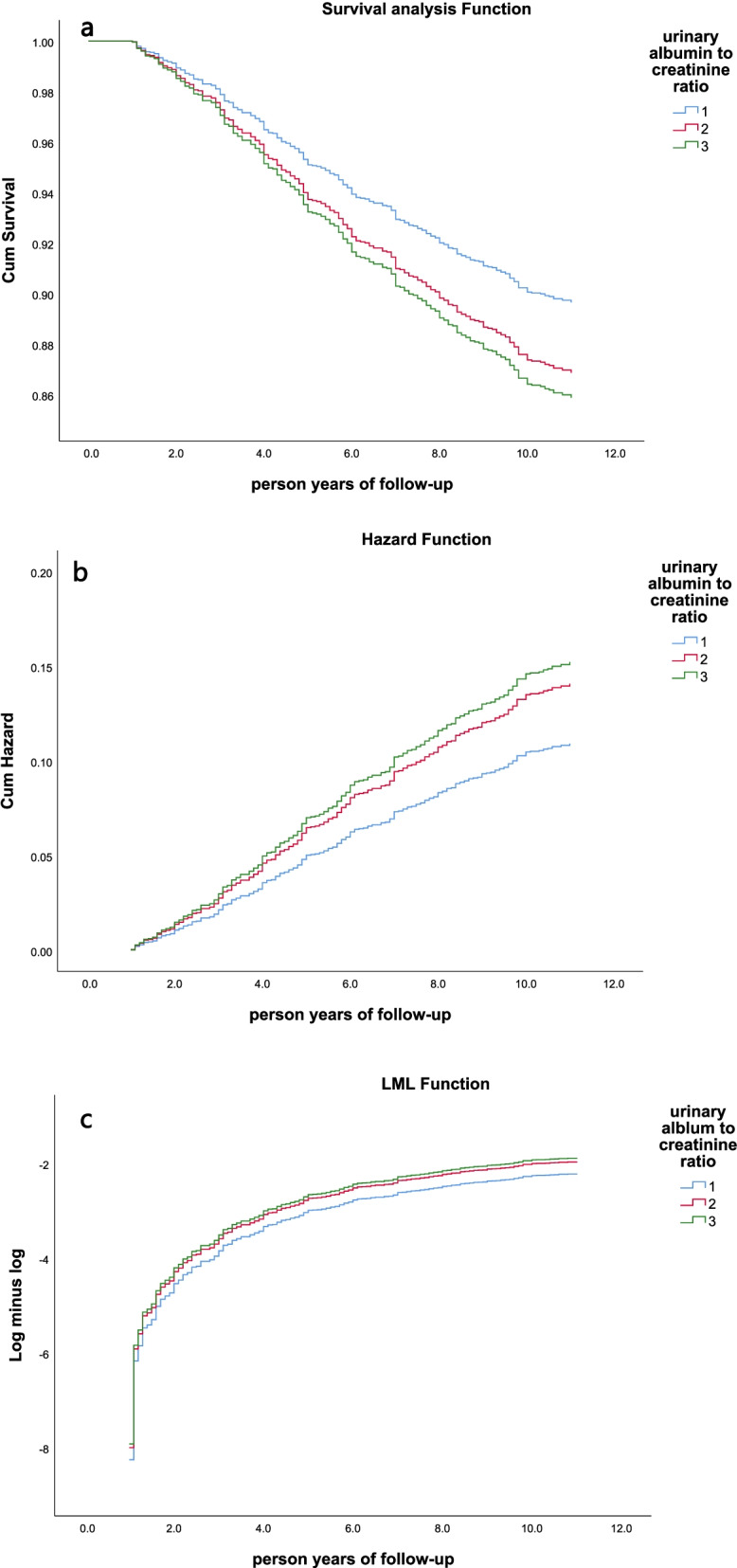
Cox proportional hazards after adjusting age, gender, height, weight, BMI, waist circumference, systolic blood pressure, diastolic blood pressure, triglycerides, blood creatinine, blood uric acid, history of coronary heart disease, history of diabetes, history of drinking. **a** Survival analysis function; **b** Hazard function; **c** LML function. Group 1 (UACR< 10 mg/g), Group 2 (10 mg/g < UACR< 30 mg/g), Group 3 (UACR> 30 mg/g)

As shown in Fig. 3, from the survival analysis curve, risk curve and LML function, it can be seen that the survival rates of the three groups are significantly different (*P* < 0.001), and the greater the UACR, the greater the risk of death in the group. In the Cox proportional hazard model, taking group 1 (UACR< 10 mg/g) as a reference, the hazard ratio (HR value) of group 2 is 1.289 times that of group 1 (*P* = 0.048). The hazard ratio (HR value) of group 3 is 1.394 times that of group 1 (*P* = 0.037). From Table 3, the risk of all-cause death in patients with high UACR is lower than the risk of all-cause death in patients with low UACR. In each influencing factor of Cox proportional hazard model, UACR has a relatively large impact on all-cause death compared with other influencing factors.

**Table 3:** Results of the multivariate Cox analyses on the risk of death by UACR levels

Variable	HR	95% CI for HR (lower)	95% CI for HR (upper)	P
**Age group (years)**	3.710	2.886	4.769	0.000
**Sex (females %)**	1.627	1.141	2.320	0.007
**BMI (kg/m**^**2**^**)**	0.859	0.658	1.120	0.262
**Height (cm)**	0.956	0.879	1.041	0.299
**Weight (kg)**	1.021	0.920	1.133	0.694
**Waist circumference (cm)**	1.042	1.021	1.063	0.000
**Systolic blood pressure (mmHg)**	1.009	1.002	1.016	0.014
**Diastolic blood pressure (mmHg)**	0.981	0.967	0.995	0.007
**Triglycerides (mmol/L)**	0.720	0.613	0.845	0.000
**Blood creatinine (umol/L)**	1.004	1.001	1.007	0.015
**Blood uric acid (umol/L)**	1.001	1.000	1.002	0.228
**History of coronary heart disease**	1.153	0.904	1.471	0.251
**History of diabetes**	1.103	0.841	1.448	0.477
**History of drinking**	1.124	0.863	1.464	0.385
**UACR**				0.045
**UACR (1)**	1.289	1.002	1.659	0.048
**UACR (2)**	1.394	1.020	1.905	0.037

UACR: Group 1 (UACR< 10 mg/g), UACR (1): Group 2 (10 mg/g < UACR< 30 mg/g), UACR (2): Group 3 (UACR> 30 mg/g)

## Discussion

In this present study, after adjusting age, gender, height, weight, BMI, waist circumference, systolic blood pressure, diastolic blood pressure, triglycerides, blood creatinine, blood uric acid, history of coronary heart disease, history of diabetes, history of drinking, taking group 1 as a reference, the hazard ratio of group 2 was 1.289 times that of group 1 (*P* = 0.048). The hazard ratio (HR value) of group 3 was 1.394 times that of group 1 (*P* = 0.037). The normal urine albumin-creatinine ratio recognized in our country is < 30 mg/g. Group 2 (10 mg/g < UACR< 30 mg/g) is a community population with a higher urine albumin-creatinine ratio within the normal range. Our results show that even if the urinary albumin and creatinine ratio is normal, if the UACR value is higher, it will still have a certain impact on all-cause death. This is the innovation of our research. Before our research, there have been reports at home and abroad that microalbuminuria (30 mg/g < UACR< 300 mg/g) can predict the risk of cardiovascular and all-cause death [22, 23, 29]. There is no doubt that UACR> 30 mg/g is proteinuria. Studies have divided proteinuria into microalbuminuria (30 mg/g < UACR< 300 mg/g) and massive proteinuria (UACR> 300 mg/g), and there is evidence that microalbuminuria can predict cardiovascular death and all-cause death [7, 30]. However, the research on the relationship between the normal high value of UACR (10 mg/g < UACR< 30 mg/g) and all-cause death is not detailed enough.

In 2020, another Chinese study showed that urinary albumin-creatinine ratio (UACR) within the normal range (less than 30 mg/g) would also increase the risk of cardiovascular disease. The optimal cut-off value of UACR for diagnosing diabetic left ventricular hypertrophy is 10 mg/g [27]. In our study to predict all-cause death, we used the same cut-off value, and the results were similar. In our study, the same cut-off value was used by us to predict all-cause death. The results are similar. The risk of all-cause death in the group with UACR< 10 mg/g is the lowest, and the results are statistically significant. The reason may be that kidney damage is a continuous process. In the process of the urinary albumin-creatinine ratio gradually increasing, the damage has already formed, which has a certain impact on the outcome of all-cause death. Therefore, the UACR range in the normal high value (10 mg/g < UACR< 30 mg/g) does not mean that it is healthy, and the damage has already occurred, which deserves more attention and research. A UK study in 2003 have shown that microalbuminuria (30 mg/g < UACR< 300 mg/g) can predict all-cause and cardiovascular disease mortality in men and women independently of other established cardiovascular risk factors. In all subjects Among them, compared with participants with normal albuminuria, individuals with microalbuminuria increased the risk of all-cause death by about 50%, and the risk of death from cardiovascular disease increased by about twice [17]. The results of our study are consistent with the study in the United Kingdom. There is a dose response relationship between the degree of proteinuria and the risk of death. However, the measurement unit range standard for the UACR used in the UK is different from that in China. In addition, unlike their research, our follow-up study time is longer, with an average of 9.87 years (18,407.3 person-years of follow-up). It makes our results more credible. Most importantly, the urine samples in their study were cryopreserved, and the measured urinary albumin to creatinine ratio may be inaccurate. Our data were all measured on-site during community surveys. A large-scale study in the Netherlands in 2002 pointed out that urine albumin excretion can predict cardiovascular and non-cardiovascular deaths in the general population [31]. The study included a population of 40,548 people with a large sample size. The results of the study showed that urinary albumin excretion increased twice, the relative risk of cardiovascular mortality was 1.29, and the relative risk of non-cardiovascular mortality was 1.12. The follow-up time of this study was only 3 years, so the mortality rate in the results was lower, and the relative risk of mortality was lower than the British study and our study.

In predicting the occurrence of cardiovascular disease, a 2005 study in the United States presented evidence that challenged the concept that UACR< 30 mg/g means normal [7]. This is the starting point for us to consider the relationship between normal UACR and all-cause death. The 2007 ESC-ESH Hypertension Management Guidelines stated: “In diabetic and non-diabetic hypertensive patients, microalbuminuria, even if it is below the currently considered threshold, has been proven to predict cardiovascular events.” [32] In the studies mentioned in the guidelines, the study population of Redon J et al. in 2002 and de Leeuw PW et al. in 2004 were mainly concentrated in the special hypertensive population [33, 34]. The study of Bigazzi R et al. in 1998, Jensen JS et al. in 2000, and Arnlöv J et al. in 2005 mainly explored the relationship between proteinuria and cardiovascular events [7, 16]. Although Gerstein HC et al. in 2001 and Jager A et al. in 1999 studied the relationship between proteinuria and death, their follow-up time was only 4.5 years and 5 years [6, 35].

Our study is the first time that groups within the range of normal urine albumin to creatinine ratio are used to predict all-cause mortality in community populations with 10-year follow-up. In terms of the pathological mechanism of the relationship between urine albumin-creatinine ratio and all-cause death, proteinuria may be an early sign of vascular endothelial dysfunction, which easily leads to the development of individual atherosclerosis [36]. Atherosclerosis and increased pulse pressure further damage vascular endothelial cells, forming a vicious circle, causing damage to the whole body. A study in Taiwan, China in 2018 also showed that there is a linear relationship between proteinuria and arterial stiffness in Chinese adults [37]. Arteriosclerosis is an important risk factor for cardiovascular and cerebrovascular diseases, which may be the pathological cause leading to all-cause death.

Compared with other studies, our study has a 10-year follow-up, a novel grouping standard, and enough community populations to prove the relationship between urine albumin-creatinine ratio and all-cause deaths in Chinese community populations. However, our study also has some shortcomings. First, most of our follow-ups are conducted through telephone consultation, and the degree of cooperation and response accuracy of the interviewed population may be biased. Secondly, in our study, 294 (13.7%) lost follow-up in 10 years. This tendency to attrition may have weakened our results. In the end, we had 1854 people included in the study, and the reduced sample size also reduced the robustness of our conclusions.

Previously, only in patients with hypertension and diabetes, doctors would pay attention to the patient’s urinary albumin to creatinine ratio, because many studies have shown that proteinuria is independently related to all-cause death. However, in the non-diabetic hypertensive population and the general community population, people pay insufficient attention to the ratio of urinary albumin to creatinine ratio. Through our research evidence, in clinical practice, doctors will pay more attention to the UACR indicator and have more choices in predicting the indicators and standards of death from all causes.

## Data Availability

The research data used to support the findings of this study are available from the corresponding authors upon request.

## Abbreviations

UACR: Urinary albumin to creatinine ratio
HR: Hazard ratio
CI: Confidence interval
CKD: Chronic kidney disease
ESKD: End-stage renal disease
CV: Cardiovascular
NIHSS: National institutes of health stroke scale
MMSE: Minimum mental state examination
ADL: Activity of daily living
CHD: Coronary heart disease
